# Sustained Recovery in a Treatment-Refractory Obsessive–Compulsive Disorder Patient After Deep Brain Stimulation Battery Failure

**DOI:** 10.3389/fpsyt.2020.572059

**Published:** 2020-11-13

**Authors:** Redwan Maatoug, Antoni Valero-Cabré, Philibert Duriez, Bertrand Saudreau, Sara Fernández-Vidal, Carine Karachi, Bruno Millet

**Affiliations:** ^1^Sorbonne Université, AP-HP, Service de psychiatrie adulte de la Pitié-Salpêtrière, Institut du Cerveau, ICM, Paris, France; ^2^Groupe de Dynamiques Cérébrales, Plasticité et Rééducation and Frontlab Team, Institut du Cerveau (ICM), INSERM 1127, CNRS, UMR 7225 and Sorbonne Université (SO), Paris, France; ^3^Institut du Cerveau et de la Moelle Epinière (ICM), CNRS UMR 7225, INSERM U 1127, Sorbonne Université, Paris, France; ^4^Laboratory for Cerebral Dynamics Plasticity and Rehabilitation, Boston University, School of Medicine, Boston, MA, United States; ^5^Cognitive Neuroscience and Information Technology Research Program, Open University of Catalonia (UOC), Barcelona, Spain; ^6^Institute of Psychiatry and Neurosciences of Paris, Unité Mixte de Recherche en Santé (UMRS) 1266 Institut National de la Santé et de la Recherche Médicale (INSERM), University Paris Descartes, Paris, France; ^7^Clinique des Maladies Mentales et de l'Encéphale, Groupement Hospitalier Universitaire (GHU) Paris Psychiatry and Neuroscience, Sainte-Anne Hospital, Paris, France; ^8^Université Pierre et Marie Curie-Paris 6, Centre de Recherche de l'Institut du Cerveau (CRICM), UMR-S975, Paris, France; ^9^INSERM, U975, Paris, France; ^10^CNRS, UMR 7225, CR-ICM, Paris, France; ^11^Centre de Neuroimagerie de Recherche de l'Institut du Cerveau (CENIR ICM), Paris, France; ^12^Neurosurgery Department, APHP, Hôpitaux Universitaires Pitié-Salpêtrière/Charles Foix, Paris, France

**Keywords:** obsessive compulsive disorder, deep brain stimulation, treatment refractory, stimulation techniques, battery failure, case-report

## Abstract

Obsessive-compulsive disorder (OCD) is a widespread chronic neuropsychiatric disorder characterized by recurrent intrusive thoughts, images, or urges (obsessions) that typically cause anxiety or distress. Even when optimal treatment is provided, 10% of patients remain severely affected chronically. In some countries, deep brain stimulation (DBS) is an approved and effective therapy for patients suffering from treatment-resistant OCD. Hereafter, we report the case of a middle-aged man with a long history of treatment-resistant OCD spanning nearly a decade with Yale–Brown Obsessive Compulsive Scale (Y-BOCS) scores oscillating between 21 and 28. The patient underwent bilateral implantation of ventral striatum/ventral capsule DBS leads attached to a battery-operated implanted pulse generator. After a 3-month postimplantation period, the DBS protocol started. Three months after the onset of DBS treatment, the patient's Y-BOCS score had dropped to 3, and he became steadily asymptomatic. However, inadvertently, at this time, it was found out that the implanted pulse generator battery had discharged completely, interrupting brain stimulation. The medical team carried on with the original therapeutic and evaluation plan in the absence of active DBS current. After 12 additional months under off-DBS, the patient remained at a Y-BOCS score of 7 and asymptomatic. To our knowledge, this is the first report that provides an opportunity to discuss four different hypotheses of long-term recovery induced by DBS in a treatment-refractory OCD patient, notably: (1) A placebo effect; (2) Paradoxical improvements induced by micro-lesions generated by DBS probe implantation procedures; (3) Unexpected late spontaneous improvements; (4) Recovery driven by a combination of active DBS-induction, the effects of medication, and DBS-placebo effects.

## Plain Language Summary

Deep brain stimulation (DBS) is an approved, effective therapy for patients suffering from treatment-resistant obsessive–compulsive disorder (OCD). Here, we report the case of a middle-aged man with nearly a decade long history of treatment-refractory OCD who, in the context of a double-blind, randomized study, underwent bilateral implantation of DBS leads in the ventral striatum/ventral capsule region. After 3 months of DBS stimulation, the patient showed a dramatic drop of OCD severity score and became stable and asymptomatic. However, investigators found out that the battery of the stimulator had emptied completely, and brain stimulation had inadvertently turned off. Importantly, the patient remained symptom-free for an additional year, in the absence of any effective DBS treatment.

## Background

Obsessive–compulsive disorder (OCD) is a widespread chronic neuropsychiatric disease characterized by recurrent intrusive thoughts, images, or urges (obsessions) that typically cause anxiety or distress. It is also associated with repetitive mental or behavioral acts (compulsions) that patients feel an urge to perform. OCD is considered the fourth most common mental disorder in developed countries, and according to the World Health Organization (WHO), represents the 10th leading cause of disability (1). Effective treatments for OCD include cognitive–behavioral therapy and serotonin reuptake inhibitors. Even when optimal treatment is provided, 10% of patients remain severely affected with treatment-resistant OCD (2). For the latter, deep brain stimulation (DBS) is the last available therapeutic option. Although the number of severe OCD treatment-resistant patients treated with DBS is still low and optimal targeting and stimulation parameters are still under debate, most studies (3) confirm that DBS constitutes an adequate treatment, with an acceptable profile of adverse effects and a global percentage of Yale–Brown Obsessive Compulsive Scale (Y-BOCS) reduction estimated at 45% and a global percentage of responders of 60%.

## Deep Brain Stimulation

DBS involves the implantation of intracranial multielectrode leads with several contacts (also referred as electrodes) used to deliver trains of electrical pulses to specific brain locations such as the subthalamic nucleus (STN) or the ventral striatum/ventral capsule (VS/VC) by means of an implanted pulse generator (IPG) (4–6). The implantation of DBS leads and the delivery of electrical current do not cause major neural damage but require brief adjustments of stimulation parameters during the postoperative period and often lifelong maintenance involving battery replacement, the repair of hardware dysfunction, and, medically, the prevention of infections. Although shown effective in treating several disorders such as Parkinson's disease (7), dystonia (8), Tourette syndrome (9), and OCD (10), the underlying mechanisms leading to DBS-mediated improvements remain unknown. Moreover, the optimal locations where to implant electrodes and the best stimulation parameters (frequency, amplitude, and the number of pulses) are still debated.

**Figure 1:**
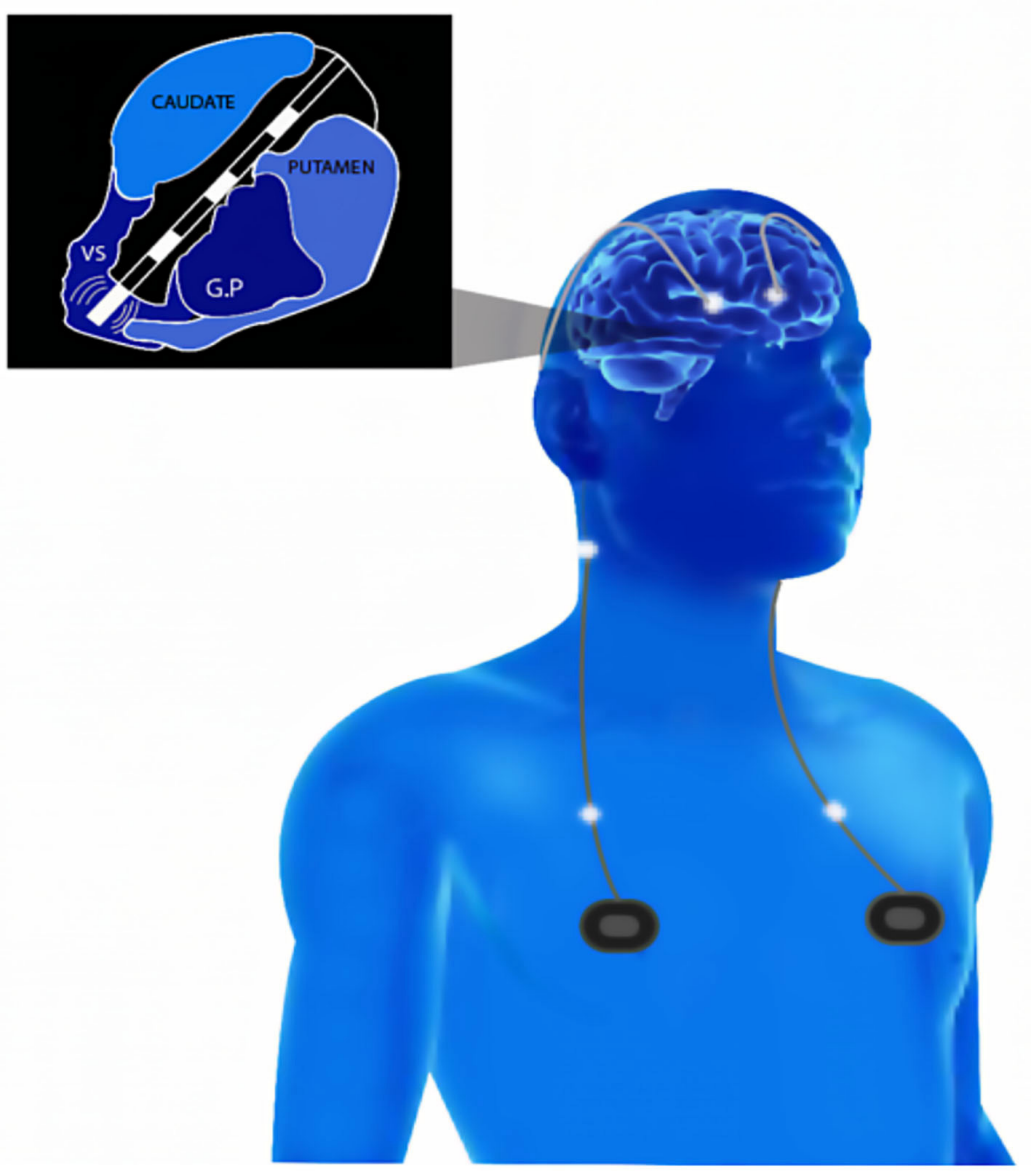
Schematic drawing of a deep brain stimulation (DBS) multielectrode implanted lead and an implanted pulse generator (IPG) on OCD patients. Patterns of electrical activity and customized parameters of frequency, intensity, and pulse duration are delivered to improve the clinical symptoms of OCD. A multielectrode lead (Medtronic Model #3391) was inserted through the internal ventral capsule (VC) so that one of its contacts (3 mm long, 4 mm interelectrode distance) reach either the ventral striatum (VS) or the subthalamic nucleus (STN). VS, ventral striatum; GP, globus pallidus.

The circuits connecting the orbitofrontal cortex (11, 12), the medial prefrontal cortex, the basal ganglia, and the thalamus are thought to be central to the pathophysiology and therapeutic response in OCD (13, 14). A widely accepted hypothesis is that the symptoms characterizing this condition are caused by an abnormally high-degree of hyperactivity in a cortico-striato-pallidal-thalamo-cortical (CSPTC) network (15, 16), which could be plausibly inhibited or functionally overridden with high-frequency DBS electrical patterns (11, 17, 18). To achieve the latter goal, DBS systems include three main components: an IPG, an intracranially implanted multielectrode lead with several contacts (or electrodes) along its bottom end, and a connection/extension cable linking the latter to the former. Electrical impulses are generated by the IPG, which is supplied in electricity by an embedded battery. This device is surgically implanted in the patient's chest subcutaneously. An extension cable running under the skin of the neck and the scalp, from the IPG through a burr hole in the skull, connects to the lead, which is implanted neurosurgically in a specific brain subcortical structure, such as the STN or the VS/VC region (see Figure 1 for details). The IPG used in DBS systems integrates a microchip, allowing neurostimulation parameters (current intensity, frequency, and pulse width) to be programmed and fine-tuned via an external portable appliance communicating wirelessly with it. Once multielectrode leads are implanted, the site of DBS neurostimulation can be further adjusted in the close vicinity of the implanted area by conveying electrical pulses to different contacts along with the lead, usually present in several 4, and activating them independently (19).

After a quick initial coarse tuning of stimulation variables, further DBS parameter adjustment takes place on average between the first 3 and 6 months [see details in (10, 11)] guided by changes in symptom severity observed in patients and measured with clinical scores. Once established, DBS parameters remain effective for the following 12 months or even longer periods. Accordingly, stimulation parameters inducing clinical benefit shown in OCD by decreases of Y-BOCS scores are maintained until further notice and kept unchanged until the end of a clinical trial follow-up period. Typical stimulation parameters for DBS in OCD may vary within the following ranges (20): Pulse frequency between 130 and 185 Hz, current power between 1 and 10 V, and biphasic pulse width between 60 and 150 ms.

## Case Report

In 2007, a middle-aged man employed as a boilermaker was admitted into the hospital (Hôpital Pitié-Salpêtrière, Psychiatry Department, in Paris, France) after a work-related accident. His jacket caught fire while he was welding a metal piece. The patient was severely burned and underwent a skin grafting intervention. After this accident, the patient developed typical OCD symptoms, according to the Diagnostic and Statistical Manual of Mental Disorders, Fourth Edition, with washing rituals, repetitive verifications, and fear of being contaminated. In 2011, his symptomatology started to increase in duration, impacting his life insidiously for ~7 h a day. In an attempt to control his symptoms, the patient was treated serially with monotherapy of fluoxetine (80 mg/day), then venlafaxine (375 mg/day), and ultimately clomipramine (225 mg/day). Nonetheless, pharmacological treatment failed to yield any sign of clinical improvement. During this period, CBT cognitive-behavioral therapy was also carried out unsuccessfully.

In 2012, the patient attempted suicide by voluntary drug poisoning. This event was explained by occupational inactivity, serious degradation of his quality of life caused by severe OCD symptoms, and increased alcohol consumption. At this time, new pharmacological therapies were attempted with a lower dose of risperidone (3 mg/day), aripiprazole (15 mg/day), and quetiapine (400 mg/day), once more, without clinical success. Subsequently, in 2015, a noninvasive transcranial brain neuromodulation treatment was also assayed via a regimen of 20 daily sessions of repetitive transcranial magnetic stimulation, delivering low-frequency patterns (1 Hz, intensity set at 120% of his resting motor threshold, for 20 min, 1,200 pulses per session) targeting the orbitofrontal cortex (21). Nonetheless, no major improvement was noticed either. Indeed, it should be noted that despite the treatments applied from 2007 to 2016, the patient's Y-BOCS remained relatively stable at high severity scores between 21 and 28 (out of a total of 40 points), a fact that attests the enduring and long-lasting nature of his OCD symptoms.

**Figure 2:**
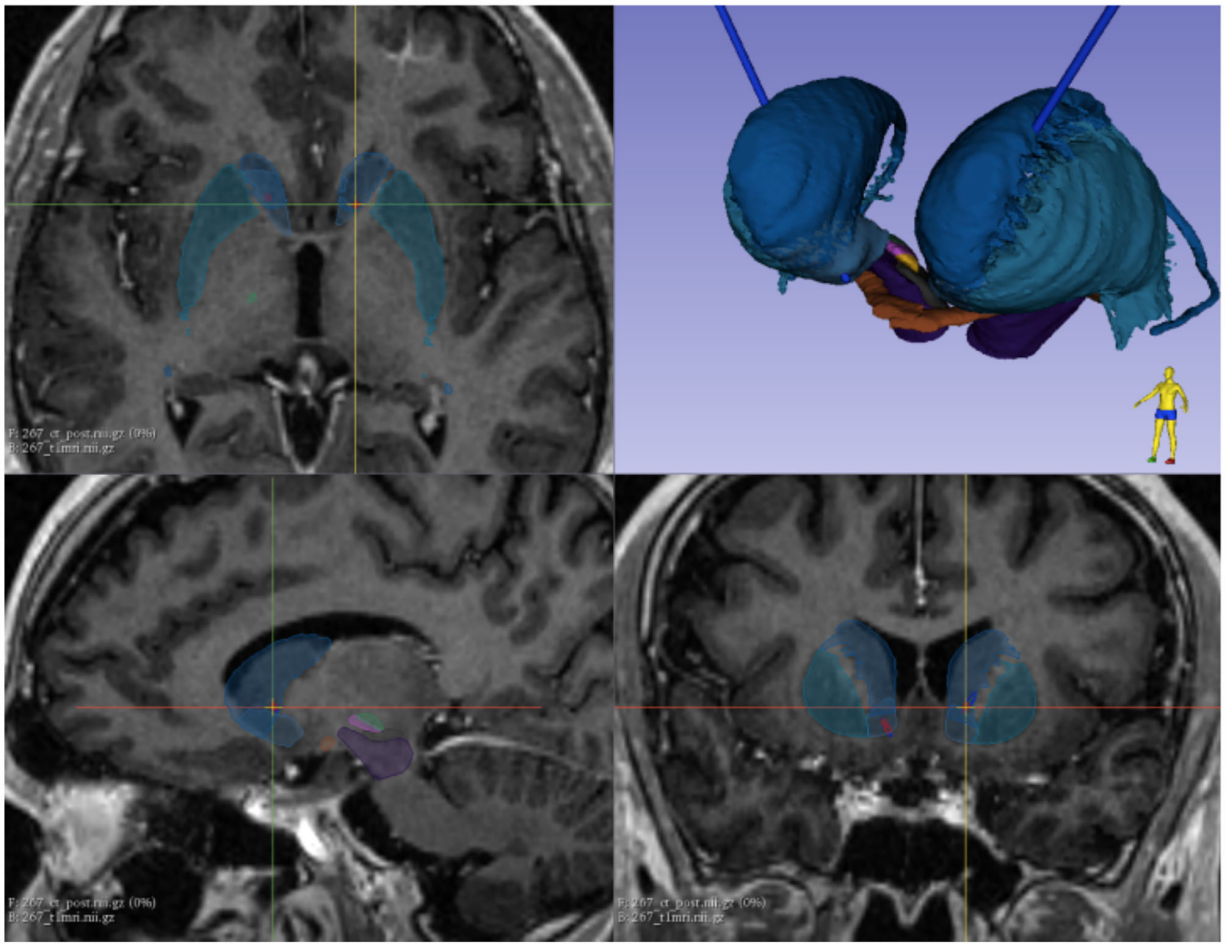
Implanted multielectrode leads were visualized using a combination of a preimplantation T1 MRI volume (without and with gadolinium injection), a T2 MRI sequence, and a validated three-dimensional histological and deformable YeB atlas of the basal ganglia. Blue, caudale nucleus; dark green, putamen; purple, substantia nigra; Pink, limbic subthalamic nucleus; green, sensorimotor subthalamic nucleus.

In 2016, the patient was included in a prospective randomized, double-blind DBS clinical trial. The design of the study included two arms; in one condition, two multielectrode leads were implanted bilaterally into the right and left VS/VC, whereas, in the other arm of the clinical trial, leads were also implanted bilaterally into the STN. Implanted multielectrodes were non-MRI compatible quadripolar stimulation leads (Model #3391, Medtronic, Minneapolis, MN, USA) made of four contacts (4.0-mm spacing between four 3.0-mm long electrodes). Target regions were identified using a combination of T1 (without and with gadolinium injection) and T2 MRI sequences and a validated three-dimensional histological and deformable YeB atlas of the basal ganglia (22). Neurosurgical trajectories were planned and selected to allow contacts in both the left and the right VS/VC regions. Implantations were performed using a Leksell frame (Elekta Instruments, Inc.) assisted intraoperatively with real-time X-ray guidance and microelectrode recordings. Implantations in our patient, targeted the right and left VS/VC site with the following Montreal Neurological Institute coordinates: right VS/VC: *X* = −8, *Y* = 34, *Z* = 4.5 (Leksell frame *X* = 91.5, *Y* = 118.5, *Z* = 113, *A* = 65, and *B* = 70) left VS/VC: *X* = 9.5 (Leksell frame *Y* = 33, *Z* = 4, *X* = 109, *Y* = 117, *Z* = 112.5, *A* = 120, *B* = 78). The final location of the four contacts at the bottom end of each multielectrode lead was semiautomatically detected on a postoperative O-Arm helical CT scan linearly registered to the preoperative T1-weighted MRI; the YeB atlas was co-registered to Figure 2 (23). As usually done in prior protocols, electrodes were left to settle for 3 months. By the end of the third month, and after a short period of parameter adjustment well-detailed elsewhere (10, 11), stimulation was set up at some fixed parameters (see later for details on stimulation parameters).

By inclusion criteria, the patient had to attest to maintaining the same pharmacological treatment during 6 months preceding lead implantation neurosurgery and throughout his participation in the DBS trial, until the end of the follow-up. Hence, 6 months prior and throughout his participation in the DBS clinical trial, our patient was on fluoxetine 60 mg/day, aripiprazole 10 mg/day, and clomipramine 75 mg/day. No changes in medication were made during this period.

After a postimplantation “off-stimulation” period of 3 months, stimulation with the IPG (Medtronic Activa^TM^ PC non-rechargeable neurostimulator) started. A set of parameters were implemented in a first stimulation protocol targeting bilaterally the VS/VC (130 Hz, 6 V, pulse width at 90 ms, delivered through lead contacts n° 3 and n° 11 for right and left stimulation, respectively). After a short period devoted to parameter adaptation and guided by short-term clinical responses, the former protocol was modified by adding the bilateral stimulation of the associative portion of the caudate nucleus. This modified stimulation protocol was then applied continuously for three additional months (130 Hz frequency, 3 V, and biphasic pulses of 120 ms width, delivered through lead contacts n° 2 and n° 10 for right and left stimulation, respectively).

Before DBS onset, by the end of the period that followed lead implantation, the patient's Y-BOCS score remained severely affected (Y-BOCS score of 22). Nonetheless, 3 months after the theoretical onset of DBS using the final set of fixed parameters reported earlier, the patient no longer complained of OCD symptoms, and his Y-BOCS fell to a score of 3. Clinically, he seemed soothed and did not feel the urge anymore for washing rituals, neither reported experiencing an insidious sense of contamination risk. The patient was euthymic; his personal interactive relationship skills had greatly improved; and he no longer consumed alcohol. Attesting such dramatic improvements, the patient even planned to resume his professional activity. Consequently, follow-up appointments were gradually spaced out, and 9 months post-lead implantation (hence after 6 months of DBS treatment), the patient was asymptomatic and stable, still with a low Y-BOCS score of 3. Nonetheless, at this time, during a routine check, the psychiatrist in charge of the study unexpectedly found out that the IPG was no longer operative because the battery had depleted completely long before. Unfortunately, we could not determine how long the battery might have been depleted by interrogating the IPG unit. The study blind was lifted, and investigators learned that the patient turned out to have been randomized to the VS/VC bilateral stimulation condition and treated with the last set of the earlier-reported parameters. In the absence of any specific complaint, and given the outstanding patient's recovery with consistent low Y-BOCS scores, in agreement with the patient, we decided not to replace the IPG. This decision was taken because it seemed unreasonable to operate the patient again considering: (1) the risk of infection or hemorrhage; (2) the risk of the anesthesia associated to the surgical procedure; (3) the risk of causing additional micro-lesions during implant removal; and (4) the risk of a recrudescence of OCD symptoms.

**Figure 3:**
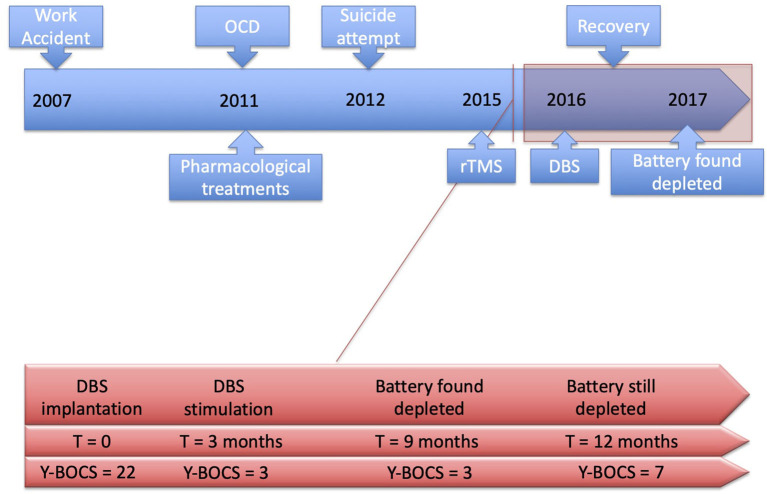
Timeline of the symptoms history and their management.

More regular medical consultations were, however, scheduled monthly, to follow the evolution of such an unexpected case. In January 2019, after a 12-month follow-up (Figure 3), the patient remained still stable and asymptomatic with a low Y-BOCS score of 7.

## Discussion

To the best of our knowledge, this is the first reported clinical case presenting evidence suggestive of DBS placebo recovery in a patient with severe OCD scores in the context of a stimulation regimen, halted inadvertently by battery depletion. Importantly, the patient had shown symptoms refractory to pharmacological and noninvasive brain stimulation treatments, spanning for nearly a decade. Hence, it is very unlikely that the achieved recovery might have been triggered “spontaneously” or could have occurred totally unrelated to the DBS procedures undergone as the ultimate therapeutic option.

Interestingly, evidence from the National Institutes of Health OCD DBS patient cohort addressing the clinical consequences of IPG battery depletion and/or DBS device failures instead of OCD improvements reported increases in Y-BOCS scores and severe neuropsychiatric and mood symptoms, together with motor restless-like sensations (24). On this basis, either a very effective induction of after-effects after a short period of DBS stimulation or a DBS mediated placebo impact should be considered as potential recovery factors in this single patient OCD case. These findings are potentially promising because they could allow psychiatrists treating medication-resistant OCD patients with DBS to design stimulation regimens that operate during restricted periods instead of being delivered chronically, as it is most often the case. These alternative approaches could also significantly reduce the number of pulses and the duration of DBS regimens (hence, the total current delivered on a given brain area), limiting battery consumption in IPG systems.

The findings here reported are coherent with several clinical cases compiled in recent publications, reporting similar phenomena on other types of pharmaco-resistant disorders treated with DBS (23–25). More specifically, the case of a single patient with Tourette syndrome implanted bilaterally in the center median–parafascicular complex of the thalamus and the limbic territory of the internal portion of the globus pallidus revealed decreases of tic severity and self-injurious behavior, after implantation surgery and before stimulation was ever applied (23). Moreover, a systematic review studied the frequency and magnitude of placebo effects in a sample of 126 Parkinson's disease patients treated with DBS (25). Outcomes showed that active DBS was more effective when preceded by a sham-DBS block than in the absence thereof, estimating the contributions of placebo-DBS as accounting for 39% of the total recovery driven by active-DBS. Finally, a study in tremor-dominant Parkinson's disease treated with DBS to the STN showed that patient's expectations about stimulation therapy modulated motor and cognitive dysfunction outcomes (26). Indeed, positive expectations enhanced the clinical impact of STN-DBS by further decreasing the magnitude of patient's tremor, whereas in contrast, negative expectations counteracted its therapeutic benefit and exacerbated some of its adverse effects. Taken together, this evidence suggests that placebo effects and/or positive expectations tied to such may contribute to improvement in DBS patients enduring symptoms of treatment-resistant diseases (e.g., Tourette syndrome and Parkinson's disease), in the absence of effective stimulation. Our single case report extends these intriguing observations to a well-characterized psychiatric condition also treated with DBS such as OCD. Additionally, it suggests that DBS placebo effects, as those here hypothesized, may have boosted the impact of a short-lasting DBS protocol, extending its effects over very long periods of time, once stimulation was inadvertently discontinued due to battery depletion.

We here conclude that placebo effects could play a role either inducing and/or ensuring the maintenance of attained recovery levels in treatment-refractory OCD patients.

On such a basis, and as suggested by the outcomes of a meta-analysis by Schruers et al. (27), it is paramount to rule out unexpected placebo improvements when assessing the outcomes of randomized clinical DBS trials in OCD populations. It is, however, important to emphasize that several facts curtail our ability to fully confirm these explanatory hypotheses. First, even if unlikely, given the enduring and chronic nature of OCD symptoms in our patient, we cannot completely rule out late-acting spontaneous recovery triggered by uncontrolled behavioral factors the patient might have been exposed to but failed to report to clinicians. Second, we cannot totally discard either that a very short period of effective DBS stimulation during the brief time the IPG battery could have been operational may have driven enduring recovery, independently of placebo influences over longer-term recovery maintenance. Third, we cannot completely rule out either the possibility of a synergistic interaction between ongoing medication at the time our patient integrated the study and a short period of DBS during a period in which stimulation could have been effective (indeed, both factors insufficient to drive recovery *per se* but boosting their individual effects when combined). Last, four pieces of information highlight the atypicality of the patient's clinical history and may argue in favor of spontaneous recovery as the cause of clinical improvements: (1) An OCD condition that occurred in a middle-age patient (the average onset age of symptoms in OCD patients included in DBS studies is ~15 years old), hence which may be potentially influenced by a higher loading of environmental and neurological influences [see (28) for details]; (2) OCD symptoms that appeared after a traumatic event in relation to a work accident; (3) A halt in alcohol consumption (which was significantly increased since 2012), around the time of Y-BOCS recovery; (4) Finally, the very rapid fall of Y-BOCS scores (from 22 to 3) in only 3 months) during DBS treatment.

Additionally, recovery from essential tremor in Parkinson's disease (29–31) before the onset of a systematic DBS regimen has also been associated with structural micro-lesions in implanted gray matter sites along the trajectory of the multielectrode (32). Moreover, the existence of DBS micro-lesions has been questioned by a study in STN-stimulated Parkinsonian patients that failed to demonstrate lasting signs of microelectrode damage in positron emission tomography imaging (33). Unfortunately, in our current case, the lack of postimplantation high-resolution MRI (note that implanted leads contained paramagnetic materials) and the absence of a thorough search for collateral clinical symptoms, both essential to attest micro-damage, preclude confirming or ruling out any of these hypotheses. Similarly, we can only speculate on the potential mechanisms by which enduring severe OCD symptoms wore off persistently in our patient. These could be explained by self-training or self-learning processes and the long-term suppression of abnormally excited CSPTC loop activity induced by DBS. Nonetheless, none of these scenarios can be properly assessed, given the lack of direct measures (other than clinical assessments via Y-BOCS scores) able to follow structural and physiological changes subtending the recovery of our patient. Indeed, a large majority of European Union-certified DBS multielectrode leads approved for human use contain non-MRI-compatible components, preventing the recording of structural and functional MRI datasets of sufficient quality. Additionally, these leads are unsuited to record local electrical signals from stimulated regions.

Optimizing multielectrode lead design would allow a better understanding of DBS modulation of brain systems. In fact, in OCD, this therapeutic approach hypothesizes frequency-specific effects along with a CSPTC network. These effects could be mediated via the modulation of local and network levels of activity and excitability and/or interregional synchrony. This is thus far difficult to be monitored jointly with high spatial and temporal resolution. The implantation of MRI compatible leads equipped with electrodes able to deliver current and also record intracranial electroencephalogram signals would enable precise localization of implanted brain sites and the identification of areas of damage at several intervals during a follow-up. Moreover, the coupling of the former with multimodal imaging (structural and functional MRI approaches, positron emission tomography–MRI technologies, and intracranial electroencephalogram/single-unit recordings) will grant new opportunities for monitoring hemodynamic, metabolic, and neurophysiological changes across DBS regimens and also assess the impact of micro-lesions caused by implanted multielectrode leads. Future OCD case studies similar to the one here reported, and controlled clinical trials implementing a new generation of leads coupled to imaging methods, will be in a better position to disambiguate sham DBS placebo effects from long-lasting clinical improvements induced by a short period of effective stimulation and/or sustained via placebo phenomena.

## Data Availability Statement

The original contributions presented in the study are included in the article/supplementary material, further inquiries can be directed to the corresponding author/s.

## Ethics Statement

Written informed consent was obtained from the [individual(s) AND/OR minor(s)' legal guardian/next of kin] for the publication of any potentially identifiable images or data included in this article.

## Author Contributions

BM, RM, and CK: Study concept and design. SF-V and BS:Data acquisition. RM, PD, and BS: Data analysis and interpretation. RM, AV-C, and BM: Drafting of the manuscript. RM and SF-V: Statistical analysis. All authors Critical revision of the manuscript for important intellectual content.

## Conflict of Interest

The authors declare that the research was conducted in the absence of any commercial or financial relationships that could be construed as a potential conflict of interest.
